# Functional Genomics of Legumes in Bulgaria—Advances and Future Perspectives

**DOI:** 10.3390/genes16030296

**Published:** 2025-02-28

**Authors:** Miglena Revalska, Mariana Radkova, Miroslava Zhiponova, Valya Vassileva, Anelia Iantcheva

**Affiliations:** 1 AgroBioInstitute, Agricultural Academy, Blvd. Dragan Tsankov 8, 1164 Sofia, Bulgaria; megi.revalska@gmail.com (M.R.); marianaradkova@abi.bg (M.R.); 2 Department of Plant Physiology, Faculty of Biology, Sofia University “St. Kliment Ohridski”, 8 Dragan Tsankov blvd., 1164 Sofia, Bulgaria; zhiponova@biofac.uni-sofia.bg; 3 Institute of Plant Physiology and Genetics, Bulgarian Academy of Sciences, Acad. Georgi Bonchev Str., Bl. 21, 1113 Sofia, Bulgaria; valyavassileva@bio21.bas.bg

**Keywords:** functional genomics studies in Bulgaria, model legumes, selected genes for functional characterization, future perspectives

## Abstract

Members of the Leguminosae family are important crops that provide food, animal feed and vegetable oils. Legumes make a substantial contribution to sustainable agriculture and the nitrogen cycle through their unique ability to fix atmospheric nitrogen in agricultural ecosystems. Over the past three decades, *Medicago truncatula* and *Lotus japonicus* have emerged as model plants for genomic and physiological research in legumes. The advancement of innovative molecular and genetic tools, particularly insertional mutagenesis using the retrotransposon *Tnt1*, has facilitated the development of extensive mutant collections and enabled precise gene tagging in plants for the identification of key symbiotic and developmental genes. Building on these resources, twelve years ago, our research team initiated the establishment of a platform for functional genomic studies of legumes in Bulgaria. In the framework of this initiative, we conducted systematic sequencing of selected mutant lines and identified genes involved in plant growth and development for detailed functional characterization. This review summarizes our findings on the functions of selected genes involved in the growth and development of the model species, discusses the molecular mechanisms underlying important developmental processes and examines the potential for the translation of this fundamental knowledge to improve commercially important legume crops in Bulgaria and globally.

## 1. Introduction

Members of the *Leguminosae* family, commonly referred to as legumes, are essential crops that provide food, animal feed and vegetable oils [1]. They strengthen global food security and agricultural sustainability through their high nutritional value and suitability for intercropping and crop rotation systems [2]. A key feature of legumes in sustainable agriculture and the nitrogen cycle is the unique ability to fix atmospheric nitrogen via specialized root nodules, where symbiotic rhizobial bacteria convert it into ammonia, the form usable by plants [3]. This mutual relationship supports the nitrogen nutrition of host plants and reduces reliance on synthetic fertilizers, mitigating environmental issues like nitrate pollution and eutrophication [4]. Beyond their ecological role, legumes produce secondary metabolites like isoflavones and plant sterols, which are valued for their biomedical and nutraceutical applications [4]. Advances in genomics and molecular biology have improved our understanding of legume–rhizobia symbiosis and enabled the development of varieties with increased nitrogen-fixation capabilities and greater stress tolerance [5].

### 1.1. Model Legumes and Genomic Advances

Over the last thirty years, two model legumes, *M. truncatula* and *L. japonicus*, have been extensively utilized for molecular genomic and physiological research [6]. Knowledge accumulated from these model legumes could serve for the improvement of related crops, in particular alfalfa (*Medicago sativa*), clover (*Trifolium pratense*) and birdsfoot trefoil (*Lotus corniculatus*). The complete sequencing of the genome of the model plant *Arabidopsis thaliana* [7] in 2000 marked the onset of a new era in plant genomics and supported worldwide research initiatives. Subsequent milestones included the publication of draft genomes for rice (*Oryza sativa*) in 2002 [8], poplar (*Populus trichocarpa*) in 2006 [9], *L. japonicus* in 2008 [10] and soybean (*Glycine max*) in 2010 [11]. The improved genome of *M. truncatula* was released in 2014 [12]. Developments in next generation sequencing (NGS) technologies have further driven progress, allowing the assembly of reference genomes for other important legume crops: chickpea (*Cicer arietinum*), common bean (*Phaseolus vulgaris*), cowpea (*Vigna unguiculata*) and groundnut (*Arachis hypogaea*) [13]. These genomic resources transform our understanding of legume biology and continue to advance breeding programs for improvement of crop varieties.

### 1.2. Functional Genomics—Tools and Applications

**Figure 1:**
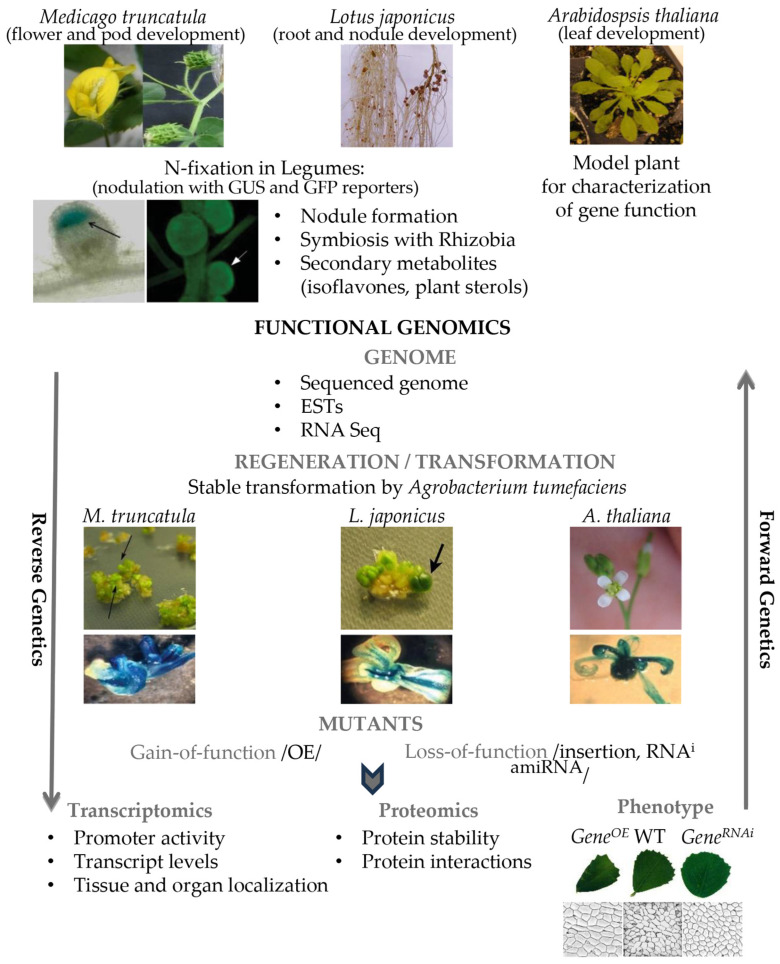
Functional genomics for characterization of unknown genes. Three model plants are used for homologous or heterologous transformation of *M. truncatula* genes derived from *Tnt1*-mutagenized population.

Among diploid annual species, *M. truncatula* stands out for its small genome size and high genetic conservation within the genus. This species has emerged as a premier model organism for genetic, genomic, molecular and functional studies, particularly promoting research on plant development and symbiotic nitrogen fixation [14]. The availability of its fully sequenced genome in Phytozome 13, along with extensive collections of mutant lines, provides powerful tools for the study of gene functions [15]. The sequenced genome reveals the coding sequences and proteins in plants, but understanding the biological roles of genes and their products requires functional genomic approaches. This strategy integrates high-throughput methods like transcriptomics, proteomics and metabolomics to decipher when and where genes are transcribed, translated into proteins, and what metabolites are synthesized. Central to functional genomic studies are mutant lines and the availability of efficient regeneration and genetic transformation systems, which allow researchers to investigate gene functions through overexpression, suppression or marker-gene fusions. Development of transgenic plants with gain- or loss-of-function mutations provides valuable insights into plant phenotypes (Figure 1).

### 1.3. Role of Transcriptomics and Proteomics for Functional Genomics

Given that many physiological and developmental processes in plants are regulated at the transcriptional level, the analysis of gene expression patterns in different organs and under specific conditions could promote the understanding of their functional roles in plant biology. Transcriptome analyses can be performed even without a reference genome, using approaches such as ESTs (Express Sequence Tags) or RNA sequencing. However, a fully sequenced genome greatly benefits the depth and accuracy of functional studies. In *M. truncatula*, transcriptomic studies have identified genes differentially expressed in various organs and in response to biotic and abiotic stresses. Special attention has been given to symbiotic nitrogen fixation, with studies focusing on dynamic processes in root nodules using plant and bacterial mutants [16]. Tools like the Gene Expression Atlas published by Benedito et al. [17,18] have shed light on gene expression patterns in root nodules and seeds, which revealed key regulatory genes and transcription factors that govern genetic reprogramming during development and differentiation.

Many legume-specific genes are predominantly expressed in nitrogen-fixing nodules, indicating their evolutionary specialization [17]. In *M. truncatula*, transcriptomic studies have further characterized gene expression responses to environmental cues and developmental stages. Research on symbiotic nitrogen fixation has elucidated the molecular mechanisms governing root nodule differentiation and function [16]. To complement transcriptomic studies, proteomics has become a key tool of post-sequencing analyses [19]. Systems biology approaches rely on well-annotated genome sequences, as annotation quality directly impacts research outcomes. The accumulation of sequencing and gene expression data in the post-genomic era requires systematic approaches to decipher gene functions.

### 1.4. Forward and Reverse Genetics and Mutant Plant Collections—Powerful Tools of Functional Genomics

Functional genomics primarily employs two strategic approaches: forward and reverse genetics, supported by large mutant collections. Among legumes, insertion mutagenesis has proven particularly powerful to discover gene functions (Figure 1). Transposable elements, especially retrotransposons (class I transposable elements) are effective for the generation of large number of insertional mutants via a copy-and-paste mechanism that integrates into host genomes [20]. The *Tnt1* retrotransposon system has been successfully implemented in *M. truncatula* (R108 and Jemalong lines) [21,22], and *LORE1* and *Tnt1* in *L. japonicus* [23,24]. During in vitro transformation, *Tnt1* actively transposes, producing *M. truncatula* lines with multiple stable inserts, ranging from 4 to 20 per regenerated plant. These insertions remain stable during the plant life cycle, and most of them are genetically independent and separable by recombination. Notably, in *M. truncatula*, *Tnt1* seems to preferentially target genes [25]. The utility of *Tnt1* has supported the creation of extensive mutant collections and enabled precise gene tagging, which has led to the identification of key symbiotic and developmental genes through forward genetics approaches [21,26]. To increase the accessibility of *Tnt1* insertion mutants, reverse genetics strategies, including PCR-based methods for the identification of flanking regions and database searches, have been developed [27,28]. These web-based and PCR-based reverse screening approaches have proven highly effective in the analysis of the *Tnt1* mutant collections.

### 1.5. Functional Genomics in Bulgaria

Building on *M. truncatula Tnt1* mutant collection created in Bulgaria, twelve years ago, our research team launched an initiative to develop a platform for functional genomic studies of legumes in the country. As part of this effort, we systematically sequenced selected mutant lines and identified genes involved in plant growth and development, which became targets for detailed functional characterization. The flanking sequences, adjacent to the insertion sites of the *Tnt1* retrotransposon (Flanking Sequence Tag, FST), can be identified by PCR-based methods, such as transposon display (TD) and two-step PCR approaches that include Inverse PCR (iPCR). The FST are stored in the publicly accessible *M. truncatula* database, which serves as a global repository for sequenced FSTs contributed by research groups working with this model plant. A subset of the *Tnt1* mutant lines developed by the Bulgarian research team is also included (https://medicago-mutant.dasnr.okstate.edu/mutant/database.php, accessed on 19 January 2025). In the framework of two projects funded by the Bulgarian National Science Fund (BNSF), part of the research focuses on cloning and functional characterization of genes that regulate plant growth and development in legumes. Analysis of the sequenced mutant lines allowed the identification of genes of interest and targets for further study [29,30]. In the mutant lines, the *Tnt1* retrotransposon was found inserted in the exons of specific genes. BLAST analysis against the databases PLAZA2.5 [31] and NCBI identified the following genes: Histone acetyltransferase *HAC1* (*Mt0g02950*); *F-box* plant like protein (*Mt2g007220*); Auxin influx carrier *LAX3* (*Mt3g072870*); Auxin response factor *ARF-B3* (*Mt5g040880*); *GRAS* family transcription factor (*Mt2g026250*) and Zinc finger CCHC-type protein (AC140549, NCBI). Based on sequencing data from the mutant lines, the above-mentioned genes were selected for cloning and functional characterization. They were also chosen for further investigation due to their role in essential processes of plant development and signaling cascades, such as secondary root formation, seed maturation, symbiotic nitrogen fixation, somatic embryogenesis and responses to environmental stress factors (Table 1). Functional genomics studies on model plants provide a basis for investigation of the complex genomes of cultivated legumes and generate valuable knowledge into their biology. The knowledge gained supports comparative genomics, an important approach for plant breeding that advances crop improvement despite the challenges posed by the large and complex genomes.

This review presents the functional characterization of selected genes in *M. truncatula* through the integration of findings from our research with the existing literature. Additionally, we outline potential future directions for the use of these functional genomics results to advance translational applications aimed at improving legume resilience, productivity and sustainability.

## 2. HAC1 as a Key Epigenetic Regulator of Plant Development and Stress Response

### 2.1. Histone Acetyltransferases—Classification and Function in Gene Regulation

Transcriptional regulation in eukaryotes, including plants, is determined not only by DNA sequence but also by chromatin modifications and remodeling. Histone acetylation, a dynamic and reversible process, can activate gene expression and impacts different biological processes (cell differentiation, growth, development) and responses to abiotic (e.g., light, temperature, drought) and biotic (e.g., pathogen attacks) stress [49]. The acetylation and deacetylation of histones are catalyzed by histone acetyltransferases (HATs) and histone deacetylases (HDACs), respectively. Generally, histone hyperacetylation correlates with transcriptional activation, whereas hypoacetylation is associated with chromatin compaction and gene repression. Research data show that plant *HATs* and *HDACs* greatly contribute to the regulation of gene expression, plant development and environmental stress responses [50,51,52]. In the review by Boycheva et al. 2014 [32], we maintain a classification that divides HATs into two main classes: HAT-A and HAT-B [53,54]. Type B HATs, located in the cytoplasm, catalyze the acetylation of free histones, whereas HAT-A proteins are localized in the cell nucleus, where they acetylate core histones of the nucleosomes, functioning as transcriptional coactivators essential for gene expression. The HAT-A class is divided into five families: GCN5-linked N-terminal acetyltransferases (GNATs), MYST (MOZ, Ybf2/Sas3, Sas2 and Tip60)-linked HATs, p300/CREB binding proteins (CBPs), and initiation factor transcription TAFII 250, which interacts with the TATA-binding protein (TBP). The Arabidopsis genome encodes five p300/CBP genes, *AtHAC1*, *AtHAC2*, *AtHAC4*, *AtHAC5* and *AtHAC12*, and two *TAFII250* genes, *HAF1* and *HAF2* [32].

### 2.2. HAC1-Mediated Regulation of Plant Growth and Development

The histone acetyltransferase *HAC1* in *A. thaliana* is homologous to the animal p300/CREB-binding proteins, which regulate transcription through acetylation of histone and non-histone proteins. Among the five identified *AtHAC* homologues in Arabidopsis, *HAC1* plays a main role in vegetative and reproductive development. Deng et al. [55] studied *AtHAC1* mutants and revealed multiple developmental defects such as delayed flowering, reduced primary root length and low fertility. Our investigation of the selected *M. truncatula HAC1* gene (*Medtr7g105660*, *Phytozome 13*) utilized stable transgenic plants with modified expression in two model legume species (*M. truncatula*, *MtHAC1^OE^* and *MtHAC1^RNAi^*; *L. japonucus*, *LjHAC1^OE^* and *LjHAC1^RNAi^*) and the reference plant *A. thaliana*. Additionally, to study the transcription of *HAC1*, plants expressing *GUS* (β-glucoronidase) and *GFP* (green fluorescent protein) reporter genes under the control of the HAC1 promoter were constructed. These transgenic plants and their progeny confirmed the expression of the *HAC1* gene in various tissues and organs with actively dividing cells. Transgenic plants with modified *HAC1* expression in all three model species showed morphological deviations, including altered plant architecture and flower morphology, which proved the gene’s role in plant development [33]. Similar morphological deviations in Arabidopsis *HAC1* mutants were reported in other studies [56,57] and in *M. truncatula* [58]. Our findings suggest that *MtHAC1* is involved in the acetylation of core histones H2B and H4, which are associated with the S phase of the cell cycle, as confirmed by transcript accumulation in plants with downregulated *HAC1* expression. The role of the gene in cell differentiation is further supported by its involvement in somatic embryogenesis. The participation of *HAT* genes in somatic embryogenesis has also been described in the studies by Daunde et al. [59] and Moronczyk et al. [52].

### 2.3. HAC1 Involvement in Responses to Abiotic Stress

Experiments with heterologously expressed *MtHAC1* in *L. japonicus* demonstrated, for the first time, that its expression in root tip cells could be modulated by treatment with the acetyltransferase inhibitor curcumin [33], showing its potential as a target for biochemical regulation and developmental studies. Our further research into the function of *HAC1* focused on its role in gene expression under abiotic stress conditions [40]. Using *A. thaliana* wild-type (WT, control) and T5 homozygous transgenic lines with overexpression (*HAC1*^OE^) and silencing (*HAC1*^RNAi^), we analyzed transcriptional and metabolite profiles under salinity and low-temperature stress. Seedlings from *HAC1*^OE^ and *HAC1*^RNAi^ transgenic lines and control lines were treated with 150 mM NaCl, and transcript levels were monitored at four time points: 0, 24, 48 and 72 h. In the *HAC1*^OE^ line, a pronounced increase in transcript levels was observed, which remained consistently high throughout the treatment period. A similar expression pattern, albeit less pronounced, was detected in the control plants. These results indicate that the studied histone acetyltransferase is involved in the response to salt stress. In contrast, the *HAC1*^RNAi^ line showed a delayed response, with a significant increase in expression observed only at 48 h compared to earlier time points (0 and 24 h). Our findings on expression profiling after salinity stress align with the data published for maize, Chinese cabbage, cotton and sugar beet, where increased expression levels of histone acetyltransferases were detected under similar stress conditions [60,61,62,63]. Application of cold stress (4 °C) for 72 h resulted in a significant elevation of transcript levels only in the *HAC1*^OE^ line. Salinity stress also induced notable changes in primary metabolism after 72 h, including elevated levels of free amino acids, sugars and fatty acids in the transgenic and WT plants. These findings are consistent with other studies, which reported changes in amino acid and sugar levels in response to salt stress in barley and rice [64,65,66,67]. Dynamic environmental changes affect plant gene expression, as histone acetylation plays an important role in the regulation of gene expression in these responses to abiotic stress. The knowledge gained from epigenetic changes in the model plant *M. truncatula* provides a valuable foundation for the improvement of legume crops under stress conditions [68].

These findings collectively demonstrate that *HAC1* functions as a master regulator of development and stress responses via control of gene expression at the epigenetic level, which shows its potential for crop improvement strategies (Table 1).

## 3. F-Box Proteins—Multifunctional Regulators of Plant Development and Metabolism

### 3.1. Molecular Mechanisms and Diverse Functions of F-Box Proteins

Plants rely on intricate regulatory mechanisms to respond to internal signals and external environmental changes, as posttranscriptional control is a major layer of gene regulation that enables rapid and precise adjustments to cellular processes. Protein degradation by the 26S proteasome is a central component of this system [69]. F-box proteins are part of enzymatic complexes responsible for the targeted ubiquitination of proteins, marking them for subsequent degradation by the proteasome [70]. These proteins ensure substrate specificity in SCF (SKP1-CULLIN-F-box) E3 ubiquitin protein ligases, which are important for the maintenance of protein turnover [71]. The expanding collection of studies sheds light on the diverse roles of F-box proteins in plant biology. They are implicated in defense responses [72], drought and salt tolerance [73,74], hormone signaling [75,76] and secondary metabolism [77,78]. F-box genes regulate key developmental processes related to organ growth [79], pollen development [80], floral development [81], leaf size control [82] and seed germination [83].

### 3.2. Conservation and Developmental Roles of F-Box Proteins Across Plant Species

Studies on F-box proteins spans a wide range of plant species, such as rice [84], maize [85], tobacco [86], *M. truncatula* [38,41,42,87], soybean [88] and *A. thaliana* [46]. The study of Boycheva et al. (2015) [42] explored the role and primary functions of a gene encoding an *F-box* protein from *M. truncatula* (*Medtr2G007220*, *Phytozome 13*). Stable transgenic plants with overexpression and downregulation, and transcriptional reporter lines for the three model species (*M. truncatula*, *L. japonicus* and *A. thaliana*), were constructed using *Agrobacterium*-mediated transformation and served as valuable tools in this research. Various analyses, including morphological, histochemical, flow cytometry and transcriptional studies, confirmed the involvement of this protein in plant growth and development, particularly in processes such as indirect somatic embryogenesis and symbiotic nodulation. The expression of this gene was detected in all plant organs and tissues, with a preference for actively dividing cells. The transgenic lines exhibited multiple developmental variations, including in root and hypocotyl growth, leaf and silique development, ploidy levels and leaf morphology, which confirmed the role of this gene in organ development. Furthermore, experiments using a root synchronization system in *A. thaliana* revealed that the transcript levels of the G2/M transition-specific gene cyclin B1:1 (*CYCB1:1*) were increased in *AtF-box*^RNAi^ lines but reduced in *AtF-box*^OE^ lines compared to control, suggesting the potential involvement of the *F-box* gene in cell cycle regulation. The data collected from the three model species pointed to a conserved role for the *F-box* gene, providing strong evidence of its functional conservation across different plants. Our findings on the involvement of the gene in diverse plant processes, cell proliferation and cell cycle control are supported by recent studies [89,90,91]. In the study by Iantcheva et al. [38], a comparative functional characterization of the *M. truncatula F-box* gene and its *A. thaliana* ortholog (*AtF-box*, *At1g10780*) was conducted using stable transgenic plants with overexpression and downregulation of the gene. The role of *MtF-box* in the leaf development of *M. truncatula* was confirmed, showing its importance in organogenesis. Protein–protein interaction analyses identified 2-isopropylmalate synthase (IPMS), a key enzyme in the biosynthesis pathway of the branched-chain amino acid (BCAA) leucine, as a common interaction partner of MtF-box and AtF-box. In both *M. truncatula* and *A. thaliana* transgenic lines with modified *F-box* expression, the content of free BCAAs (leucine, isoleucine and valine) was measured and compared to control plants. Lower BCAA amounts were detected in *F-box* overexpression lines, whereas significantly higher levels were observed in lines with *F-box* downregulation. These results underscore the role of the *F-box* gene in the regulation of leucine homeostasis, which is important for plant growth and development (Table 1).

### 3.3. F-Box Proteins in Root Development—From Model Plants to Crop Applications

The study by Zhiponova et al. [46] focused on the *A. thaliana* F-box gene (*AtF-box*) and identified it as an important player in early root development. Expression analyses in published datasets, as well as in *AtF-box^OE^* with *AtF-box-GFP*, demonstrated that *AtF-box* is actively expressed in primary and lateral roots. Knockdown of *AtF-box* expression in *AtFbox^amiRNA^* root tips caused defects in distal stem cells near the quiescent center (QC), leading to disrupted cell division patterns and premature exit from proliferation and slower cell division progression in the root meristem. Consequently, downregulation of *AtF-box* resulted in reduced root growth. Conversely, in *AtF-box^OE^* lines, cell division activity in the root meristem was maintained for an extended period, enhancing the root growth rate. Our investigation of the *F-box* gene confirms its conserved functions in the model plants *M. truncatula*, *L. japonicus* and *A. thaliana*, with potential applications for the improvement of economically important crops. Monitoring *F-box* expression and its effects on root growth might improve agronomic traits (yield and stress resilience). The role of the F-box gene in the regulation of leucine and BCAA homeostasis underscores its importance for plant growth and nutritional applications. As BCAAs are synthesized only by plants, they are vital for livestock and human dietary supplements. The conserved functions of F-box proteins across model and crop species, particularly in root development and BCAA metabolism, position them as promising targets for the improvement of crop growth, stress tolerance and nutritional value.

## 4. Auxin Influx Carrier Protein *LAX3*

### 4.1. Auxin Transport Mechanisms and LAX3 Function

Plant hormones are among the most important elements driving plants’ ability to adapt to different environmental conditions. Despite being produced in low concentrations, they are highly responsive to changes in the environment and act as signaling molecules. Among the phytohormones, auxins play a dual role, regulating plant growth and development and their response to abiotic stress. Auxin transport in plants occurs over both long and short distances. Long-distance transport is fast and non-polar, performed via the phloem, whereas short-distance transport is slow and polar, from cell to cell. The polar, active cell-to-cell transport of auxin is mediated by three main classes of transporters: AUXIN RESISTENT 1/LIKE AUX1 (*AUX/LAX*) influx carriers [92,93], PIN-FORMED (PIN) efflux carriers [94] and ATP binding cassette B/P-glycoprotein/Multi-Drug-Resistance (ABCB/MDR/PGP) transporters [95].

### 4.2. MtLAX3 Expression and Development Regulation

In previous studies, the *LAX3* gene from *M. truncatula* (Medtr3g072870, Plaza 4.5, *MtLAX3*), encoding an auxin influx carrier transmembrane transporter, was successfully cloned and subsequently introduced into the model plants *M. truncatula*, *L. japonicus* and *A. thaliana* to study its expression, localization and function. Stable transgenic plants with overexpression (*MtLAX3*^OE^) and downregulation (*MtLAX3*^RNAi^) of *MtLAX3* were generated, along with transcriptional reporter lines, using Agrobacterium-mediated transformation [39]. The function of *MtLAX3* was studied in stable transformants for the first time, as previous investigations of this gene were limited to ‘composite’ transgenic plants of *M. truncatula* obtained by *A. rhizogenes*-mediated hairy roots transformation [96]. The obtained results greatly enhanced our understanding of the function of the *MtLAX3* gene in model legumes and the potential for the application of this knowledge to crop species. Stable transgenic plants of *M. truncatula*, *L. japonicus* and *A. thaliana* were characterized by histochemical, transcriptional and phenotypic analyses [39]. The expression pattern of *MtLAX3* was established in different plant tissues and organs and confirmed in plant growth and development. Expression analysis using *GUS* and *GFP* reporter genes demonstrated that *MtLAX3* is transcriptionally active during symbiotic nodulation and indirect somatic embryogenesis. Auxin is a key regulator of somatic embryogenesis and a crucial trigger for the acquisition of embryogenic competence in explant cells [97]. In papaya, the auxin influx transporter genes (*CpLAX1*, *CpLAX2* and *CpLAX3*) are primarily associated with somatic embryo development during the torpedo and cotyledonary stages. These genes are also expressed in later expression in leaves and roots, particularly in the primordia and regions, where the vasculature develops in later stages [98]. The accurate expression of *MtLAX3* is essential for root growth and development, lateral root emergence and normal leaf morphogenesis. Auxin carriers such as *PIN6* and *LAX3* maintain the auxin flux to support apical dominance and affect plant architecture. In almond scion trees, for example, these carriers help to shape plant architecture by redistributing the auxin transport, which is influenced by rootstock genotype [99]. Upregulation or downregulation of *MtLAX3* and its orthologs caused abnormal phenotypes and developmental alterations in transgenic lines of all three investigated model plants. Overexpression of *MtLAX3* in both model legumes resulted in plants with more powerful and well-developed architecture, including an increased number of secondary root branches and root nodules, larger leaf size, bigger seed pods and higher seed production. The role of this gene in the increase in nodule formation and arbuscular mycorrhiza in *M. truncatula* has been confirmed by Schnabel et al. [100]. Roy et al. [101] demonstrated the involvement of *MtLAX2*, a functional analogue of *AUX1*, in nodule organogenesis. Further, Gonzales-Hernandes et al. [102] has shown that 2-oxoglutarate negatively affects lateral roots formation, whereas enhanced expression of *LAX2* and *LAX3* was observed in roots growing under sucrose and glucose sources.

### 4.3. MtLAX3 Role in Phosphate Stress Response

The ability of *M. truncatula* plants with modified auxin transport (*MtLAX3*^OE^ and *MtLAX3*^RNAi^) to mitigate the effect of phosphate (Pi) starvation and excess was described by Revalska and Iantcheva [47]. Along with this, the interaction between auxin and strigolactones, both of which play important roles in phosphate signaling, was evaluated under the same conditions. For the study, T1 seeds from *M. truncatula* lines with *MtLAX3*^OE^ and *MtLAX3*^RNAi^ constructs, along with transcriptional reporters and WT plants, were grown on basal MS0 medium supplemented with either 1 μM Pi (deficiency) or 2 mM Pi (excess) for 21 days. Three independent lines from every transgenic construct were analyzed to evaluate alterations in phenotype, main root length (MRL), lateral root number (LRN) and relative expression levels of *MtLAX3*, *MtMAX2* (a strigolactone signaling component) and *MtMAX3* (a strigolactone biosynthetic gene). Drastic changes in root architecture were observed in the transgenic and WT plants under extreme conditions of phosphate deficiency and excess. The *MtLAX3*^OE^ lines showed remarkable plasticity in surviving phosphorus starvation, characterized by the significant increase in LRN and elevated relative expression levels of *MtLAX3*, *MtMAX2* and *MtMAX3*. These findings highlight the potential of *MtLAX3*^OE^ lines to adapt to challenging nutrient conditions and the intricate role of auxin–strigolactone crosstalk in phosphate signaling pathways. It is hypothesized that *MtLAX3* activates the phosphate cascade by facilitation of auxin transport across the cell membrane. Phosphorus deficiency is likely to increase *MtLAX3* activity, which in turn stimulates secondary rooting and inhibits main root growth. Strigolactones have been confirmed to act in concert with auxin in shaping root architecture. Han et al. [103] revealed this synergistic interaction, demonstrating the role of strigolactones in the regulation of plant architecture by the inhibition of lateral branch growth in *Quercus mongolica* seedlings. Jiang et al. [104] further illustrated the role of phytohormonal crosstalk in phosphate accumulation, showing that the endophytic fungus *Phomopsis liquidambaris* (B3) increases phosphate uptake in peanuts (*A. hypogaea* L.) under Pi conditions. The results revealed that, under low Pi conditions, B3 significantly increased the concentrations of key phytohormones like IAA, gibberellins (GAs) and cytokinins (CKs) in peanuts. Similarly, Revalska and Iantcheva [48] investigated the effect of 2,4-D application on *M. truncatula* plants with modified auxin transport and its impact on root phenotype under Pi-starvation or excess. The expression levels of *MtLAX3* and *MtMAX2* were measured before and after 2,4-D treatment. In the study, transgenic and WT plants were grown under the previously described conditions but for 14 days before 2,4-D was added to the medium. Phenotypic and biometric analyses showed drastic changes in root architecture of the transgenic and WT plants exposed to extreme Pi conditions. Under low Pi concentrations, MRL was significantly reduced in all the transgenic and WT plants after 14 days treatment. In contrast, high Pi content led to only minor reductions in MRL over the same time period. Neither Pi-starvation nor Pi-excess affected the LRN in the *MtLAX3*^OE^ and *MtLAX3*^RNAi^ lines or WT compared to normal conditions. The additional 7-day treatment with 2,4-D significantly stimulated LRN in both the transgenic and WT plants. Treatment with 2 mM Pi combined with auxin was able to rescue the phenotype of the *MtLAX3*^RNAi^ lines. All the transgenic lines and WT plants demonstrated high plasticity in response to phosphorus stress, following 2,4-D application, evidenced by a significant increase in LRN and elevated expression levels of *MtLAX3* and *MtMAX2* in roots. The application of 2,4-D activated *MtLAX3*, which subsequently triggered *MtMAX2* expression, which led to increased secondary root formation and inhibition of primary root growth. These findings advance the understanding of *MtLAX3* function in legumes, which contributes not only to the optimization of the growth and development of important crops but also to improving our knowledge of plant responses to nutritional stress factors.

### 4.4. LAX3 Involvement in Abiotic Stress Responses

In recent years, there has been a considerable increase in studies focusing on the identification and expression of genes across the entire genome. It was shown that some auxin transporter genes are involved in the response to different stress conditions [105]. The expression profiles of *AUX/LAX* genes under different stressors have been characterized in several plant species, including maize (*Zea mays* L.) [106], soybean [107], potato (*Solanum tuberosum*) [108] and Chinese hickory (*Carya cathayensis* Sarg.) [109]. In tomato, overexpression of *SlWRKY3* confers salinity tolerance, reducing oxidative stress and proline content compared to wild-type plants. Interestingly, these plants also showed an increased expression of *LAX3* genes and higher drought tolerance [110]. The essential role of *LAX3* genes as auxin transporters in cells makes them critical regulators of plant growth and development. Additionally, the role of *MtLAX3* in the response to nutritional stress, particularly phosphorus starvation, has been confirmed. All the collected information provides a foundation for future research linked to the investigation of the involvement of *LAX3* genes in abiotic stress responses. In the framework of the project “Investigating the role of *LAX3* genes in the response to abiotic stress in the model legume *M. truncatula* and the crop plants soybean and tomato (*Solanum lycopersicum*)” (KP-06 H86/3, funded by BNSF 2024), the primary objective is to elucidate the role of the auxin transporter *MtLAX3* in responses to salinity and drought stress in the model plant *M. truncatula* and the crop plants soybean and *S. lycopersicum* (tomato). The research will track the expression patterns of *MtLAX3* and its orthologs in soybean and tomato under both stress conditions. Morphological, physiological, biochemical and metabolic changes in the growth and development of the studied plants will be monitored following the induction of salt and drought stress. Understanding the functions of *LAX3* gene, which is critical for the overall growth and development of both model and crop species under abiotic stress, will provide valuable knowledge for the improvement of stress tolerance in economically important legume crops, such as soybean, and their adaptation to adverse environmental conditions.

The main role of *LAX3* in auxin transport, plant development and phosphate stress adaptation, particularly via its interaction with strigolactone signaling, makes it a valuable target for the optimization of plant architecture and nutrient stress tolerance (Table 1).

## 5. The Versatile Role of Auxin Response Factor ARF-B3

### 5.1. ARF-B3 as a Key Regulator in Auxin-Mediated Gene Expression

In plants, gene expression in response to auxin is regulated by a family of transcription factors known as Auxin Response Factors (ARFs), which function as either activators or repressors. ARF proteins bind to auxin response elements (AuxREs) in the promoters of auxin-responsive genes. The function and expression pattern of an auxin response factor from *M. truncatula* (*Mt5g040880*, PLAZA3.0 Dicots, *Medtr5G040880* PLAZA4.5, *MtARF-B3*), containing DNA-binding pseudobarrel and B3 domains, were investigated in the three model species—the legumes *M. truncatula* and *L. japonicus*, and *A. thaliana*. For *M. truncatula*, stable transgenic plants with *MtARF-B3* overexpression (OE), RNA interference (RNAi)-mediated downregulation and transcriptional reporter lines were generated [34]. In addition, *MtARF-B3* was heterologously overexpressed in *A. thaliana* and *L. japonicus*. RNAi technology was employed to downregulate orthologue genes of *MtARF-B3* in *A. thaliana* and *L. japonicus*, resulting in knockdown lines. The tissue-specific expression pattern of *MtARF-B3* was studied using transcriptional reporter plants carrying *GUS* and *GFP* reporter genes, fused to the *MtARF-B3* promoter sequence [35,36].

### 5.2. Role of MtARF-B3 in Development and Fertility

Phenotypic and morphological evaluations, quantitative real-time polymerase chain reaction (qRT-PCR) and histochemical GUS assays were employed to analyze the function and expression pattern of *MtARF-B3* during somatic embryogenesis, and tissue and organ development, in the three model species. In *M. truncatula*, *MtARF-B3* was shown to play a key role in overall plant growth and development, root architecture modeling, seed development and fertility [34]. Similarly, Shi et al. [111] reported the involvement of the ARF subfamily in shoot development. Detailed histochemical and transcriptional analyses revealed *MtARF-B3* expression during various stages of somatic embryogenesis, organogenesis and symbiotic nodulation. Barreto et al. [112] performed transcriptional profiling of the ARF subfamily, including B3-type transcription factors during the in vitro induction of somatic embryogenesis in *M. truncatula*, confirming their important role in this process. The induction of *MtLEC2*, *MtFUSCA3* and *MtABI3* genes during somatic embryogenesis suggests their potential as biomarkers for in vitro regeneration and improvement of somatic embryogenic rates in legumes and other recalcitrant crops. In *M. truncatula*, *MtARF-B3* was expressed predominantly in the leaf vascular system [34], which aligns with the reported role of ARFs in vascular tissue differentiation [113]. Expression analysis showed a strong GUS signal in the root vascular system, developing lateral root primordia and the vasculature of mature nodules, which suggest that *MtARF-B3* might be involved in both lateral root development and nodulation. The OE lines exhibited a highly branched root system and a greater number of nodules compared to the WT plants. In contrast, RNAi-mediated downregulation of *MtARF-B3* resulted in dramatic alterations in root architecture, including reduced root length, decreased root branching and fewer nodules than WT plants. In addition, strong *MtARF-B3* expression was observed in stamens and pollen grains in *M. truncatula*, suggesting a potential role in the fertility. RNAi-mediated downregulation of the gene caused significant reduction of flower number, irregular flower morphology and sterility in plants.

### 5.3. Comparative Studies in L. japonicus and A. thaliana

Similar effects to *MtARF-B3* overexpression and downregulation were observed in *A. thaliana* and *L. japonicus*. In *L. japonicus*, *GUS* reporter gene expression was localized in callus tissue and at all stages of somatic embryogenesis, confirming the involvement of *MtARF-B3* in somatic embryo development. In roots, the GUS signal was detected in the root vascular system, secondary roots, root meristem and nodules. Unlike in *M. truncatula*, the GUS signal in leaves was localized as a spotted expression in the growing regions of the leaf petiole. This suggests that *ARF-B3* may contribute to the regulation of leaf blade expansion in *A. thaliana* and *L. japonicus* [35,36]. Phenotypic analysis of *L. japonicus* plants with OE or downregulation showed drastic alterations in root morphology. RNAi knockdown lines exhibited severely reduced length and tripod-like root architecture. These changes were accompanied by a significant reduction in nodule number compared to WT plants, with knockdown of *LjARF-B3* identified as the primary cause of these root phenotypes. The overexpression of two auxin-responsive genes, *NnARF17* and *NnARF18*, from Indian lotus (*Nelumbo nucifera*) in *A. thaliana* similarly enhanced root development and improved stress tolerance in transgenic plants [114]. In *L. japonicus*, knockdown of *LjARF-B3* led to abnormal pod development and sterility with no seed production observed. Practically, silencing *LjARF-B3* was confirmed as the primary reason for sterility in *L. japonicus* plants [36].

### 5.4. Implications for Growth and Development in A. thaliana

The involvement of *ARF-B3* in somatic embryo development in *A. thaliana* was also confirmed [35]. During postembryonic development, gene reporter activity was restricted to the epidermal layer of the primary root and the base of emerging lateral root primordia but was absent in the meristematic zone of these primordia. Investigation of root growth dynamics in *A. thaliana* showed faster growth in OE lines, resulting in well-developed final root architecture. These lines were characterized by larger serrated leaves and similar flowering times to the WT and, in some cases, the presence of numerous well-developed trichomes on the leaf surface. Approximately 60% of the RNAi-mediated knockdown mutants failed to survive in soil conditions, likely due to their shortened root systems and serrated leaves. The surviving RNAi plants exhibited slower growth, were smaller in size and matured with delayed flowering compared to the WT plants. The mature RNAi plants had shorter stems, reduced fertility and decreased seed production. Knockdown of *AtARF-B3* often resulted in lethality, and the plants displayed partially or completely sterile flowers and extremely low seed production. The few seeds obtained were minute, resembling dust, and were non-viable, unable to sprout [35]. The essential role of *ARF17* (orthologue gene of *MtARF-B3*) in anther development and pollen formation in *A. thaliana* was demonstrated by Wang et al. [115]. Similarly, pMtARF-B3::GUS expression in *A. thaliana* reporter lines showed spotted localization in the growing regions of the leaf petioles, consistent with observations in *L. japonicus* transcriptional reporter plants expressing the *MtARF-B3* promoter sequence [36]. In OE lines, giant pavement cells were observed in the tip and the base regions of the leaf epidermis, while RNAi lines displayed only single giant cells in the leaf tip region. The results suggest that both overexpression and downregulation of *ARF-B3* disrupt normal cell division patterns in leaves, likely altering the expression of genes regulating cell division and expansion. Changes in hypocotyl length in lines with modified *ARF-B3* expression further indicated its role in plant growth and development. Notably, *ARF10*, *ARF16* and *ARF17*, which are targeted by mir160, have been shown to modulate hypocotyl elongation in response to light, brassinazole (BRZ, a BR biosynthesis inhibitor) or paclobutrazol (PAC, a GA biosynthesis inhibitor) in Arabidopsis [116]. The conserved functions of *ARF-B3* across *M. truncatula*, *L. japonicus* and *A. thaliana* in the regulation of key developmental processes, from root architecture to fertility, underscore its fundamental importance in plant growth and its potential value for crop improvement strategies (Table 1).

## 6. Exploring GRAS7—A Multifunctional Transcription Factor

### 6.1. GRAS7 in Genetic Engineering and Expression Studies

As part of our experimental work, we developed an efficient in vitro regeneration protocol for Agrobacterium-mediated transformation of cell suspension cultures, resulting in stably transformed and fertile *M. truncatula* plants [117]. Cell suspension culture is a versatile tool for the study of multiple physiological, biochemical, cellular and molecular traits in plants. Transformation experiments were performed with a vector carrying two reporter genes, *GUS* and *GFP*, both under the control of endogenous gene promoters from the GRAS transcription factor (named after GD3BERELLIC ACID INSENSITIVE, GAI; REPRESSOR OF GA1, RGA; and SCARECROW, SCR; *Medtr2g026250*, PLAZA 4.5 Dicots). The activity of the *GUS* reporter gene, driven by the GRAS promoter, was observed in the roots, leaves and stems of in vitro-derived plants, demonstrating successful promoter activity and gene expression in multiple tissues [117]. The genome-wide identification and characterization of GRAS family genes from *M. truncatula* were performed in studies by Song et al. [118] and Zhang et al. [119]. The gene we selected from the *Tnt1 M. truncatula* mutant collection was identified as *GRAS7*. In a subsequent study, the expression pattern of the *MtGRAS7 TF* was investigated by functional GUS assays in *M. truncatula* [37]. The activity of the *GUS* reporter gene was detected in various tissues and organs of T1-generation transcriptional reporter plants, both before and after *Rhizobium* inoculation and under salinity stress.

### 6.2. Role of MtGRAS7 in Root Development and Nodulation

Histochemical analyses were conducted using GUS-positive *MtGRAS7* transcriptional reporter plants grown hydroponically. Root samples were collected at six different time points following *Sinorhizobium meliloti* inoculation: 0 h, 6 h, 16 h, 144 h (6 days), 7 days and 50 days. In young plants, prior to *Rhizobium* inoculation, the GUS signal was detected in the vascular system of secondary root branches and the leaf petiole but was absent in the meristematic and elongation zones of the primary root cap. Six hours post-inoculation, a weak GUS signal was observed in the elongation zone of the primary root and lateral roots. In larger lateral roots, the signal appeared as a scapula shape and began forming a ring. By 16 h, gene expression was localized in the elongation and meristematic zones of the root tip and the root vascular system. Six days post-inoculation, the ring-shaped expression pattern was well-formed, and, by 7 days, *GUS* expression was detected in infection threads formed in the root hairs of transcriptional reporter plants. These threads exited the root hair cells and progressed toward the cortex. A few days later, the threads ramified into dividing cells of the nodule primordia. Notably, *MtGRAS7* expression was observed in small vesicles trafficking trough the root toward root hairs. Using light microscopy, GUS activity was identified in the lateral root primordia and the meristematic zones of the root nodules. The obtained results suggest that *MtGRAS7* expression is induced in response to bacterial infection. In mature plants, the *GUS* reporter gene under the control of the *MtGRAS7* promoter was expressed in actively dividing zones and the vascular system of roots and leaves, including the petiole, root tip, secondary root primordia and root columella [37]. These findings confirm the role of *MtGRAS7* in plant development, including nodule formation. Strong *MtGRAS7* expression was observed in the vasculature and meristematic zones of the induced nodules.

### 6.3. Role of MtGRAS7 in Abiotic Stress Responses

The expression pattern of the *MtGRAS7* TF, fused to the *GUS* reporter gene, was monitored before and after salinity stress. Transcriptional reporter plants were transferred to hydroponic containers containing 750 mL of commercial growth solution supplemented with 0, 50 and 100 mM NaCl. Samples of roots, leaves and nodules were collected at 0, 24, 48 and 72 h for GUS assays. Before and after salt exposure, GUS signals were detected in the root tips, secondary root branches, root nodules and leaf epidermal cells. Strong expression was particularly evident in the leaf regions where injury occurred. A notable difference in GUS signal intensity in the injured leaf areas was observed 24 h after exposure to 100 mM NaCl. By 48 h, *MtGRAS7* expression in the meristematic zones of the root nodules was stronger compared to the control plants. However, the intensity of GUS staining started to decrease after 72 h. Localized or point-specific expression of *MtGRAS7* was also observed in the roots, leaves and nodules following stress induction. Higher salt concentrations appeared to activate *MtGRAS7* expression, supporting its role in the regulation of plant responses to abiotic stress. To further examine the effects of salinity stress, the roots and leaves of *MtGRAS7* transcriptional reporter plants treated with low NaCl concentrations for 72 h were examined using light microscopy. Point-specific expression was observed in the epidermal cells of roots and leaves. In some root cells, small blue-stained vesicles were visible, which appeared to be actively transported out of the cells. Following the induction of salinity stress, the number of these vesicles increased dramatically. In roots, vesicles showed cellular localization, often arranged in rows or clustered in larger spherical structures. Occasionally, single vesicles detached from the roots and moved toward the root hairs. Similar structures were observed in leaves, where the vesicles appeared either as single beads or tightly packed in epidermal cells [37]. Revalska et al. [43] investigated *MtGRAS7*, a member of the PAT subgroup, to study its role in *M. truncatula*. Using stable transgenic lines with *MtGRAS7* overexpression (*MtGRAS7*^OE^) and RNAi-mediated downregulation (*MtGRAS7*^RNAi^), they compared the phenotypes to wild-type (*M. truncatula* WT plants) controls. The study represented the first functional analysis of *MtGRAS7* in stable transgenic *M. truncatula* plants. The role of this gene was examined under various abiotic stresses—drought (350 mM mannitol), salinity (150 mM NaCl) and cold (4 °C). Relative transcript levels of *MtGRAS7* were measured in the leaves and nodules at different time points during stress exposure and subsequent recovery. As expected, gain-of-function *MtGRAS7*^OE^ lines demonstrated enhanced growth and development during stress and recovery periods. Transcript levels were higher in *MtGRAS7*^OE^ lines and lower in *MtGRAS7*^RNAi^ lines compared to the WT plants. These molecular differences were reflected in phenotypic variations between the genotypes.

The OE lines developed a massive root system with multiple secondary branches and scattered nodules, whereas the RNAi and WT plants displayed long, thin and sparsely branched roots. Genotypic differences were also observed in flowers, pods and seed traits. Interestingly, the *MtGRAS7*^RNAi^ plants produced a higher number of seeds with greater weight compared to the OE lines. However, the seed quality in the OE lines was compromised, with more than 50% of the seeds being flaky and exhibiting poor germination rates. These findings suggest that *MtGRAS7* upregulation may negatively impact seed development, though the underlying mechanisms remain unclear. The potential negative correlation between *MtGRAS7* overexpression and seed formation warrants further investigation to elucidate the regulatory mechanisms involved. Overall, the results highlight the diverse and stage-specific roles of *MtGRAS7* throughout the plant life cycle. Previous studies by Ma et al. [120] and Xu et al. [121] have demonstrated the involvement of *GRAS* genes in abiotic stress responses, such as cold, drought, salt and heat. In the study by Revalska et al. [43], *MtGRAS7* was specifically analyzed under drought stress induced by mannitol and salinity stress simulated by NaCl. In general, drought-induced changes in *MtGRAS7* expression were less pronounced in the leaves, except in the OE background, where upregulation was observed at the 24-h time point. In contrast, *MtGRAS7* expression in nodules showed an initial upregulation at 24 h in all three genotypes (*MtGRAS7^OE^*, *MtGRAS7^RNAi^* and WT), followed by subsequent downregulation. The response to salinity stress was more pronounced, with significant upregulation of *MtGRAS7* expression in the leaves and nodules across the transgenic and WT plants after 24 h of treatment. In the leaves, a second peak of the expression was observed at 72 h of treatment, indicating a sustained response to salinity stress. Following exposure to salt and drought stress, the plants were transferred to greenhouse conditions for further analysis of growth and seed production. The *MtGRAS7*^RNAi^ transgenic lines were unable to survive the applied stresses, resulting in complete mortality. In contrast, the *MtGRAS7^OE^* plants exhibited higher stress tolerance and greater plasticity compared to the WT plants. Drought stress had a more severe impact on the plants than salinity stress. Despite this, the *MtGRAS7^OE^* lines showed improved seed quantity and quality compared to the RNAi lines. Interestingly, the abiotic stress appeared to have a positive effect on seed production in the OE lines, suggesting that overexpression of *MtGRAS7* may enhance reproductive resilience under adverse environmental conditions. The involvement of *MtGRAS7* in the response to cold (4 °C) stress was also confirmed. In leaf samples, the gene was clearly upregulated 72 h after cold exposure and remained elevated during the recovery period. Based on survival rates and seed production, cold stress positively affected overexpression of the gene and seed yield. Huang et al. [122] revealed the critical role of the *GRAS* gene family in cold stress tolerance in white clover (*Trifolium repens* L.) in response to cold stress through genome-wide analysis. RNA-seq and qRT-PCR experiments demonstrated early or intermittent upregulation of *GRAS* genes under cold stress, suggesting their potential importance in cold tolerance mechanisms. Probably, the structural diversity of *GRAS* TFs likely contributes to their diverse functions across different plant species, influencing processes such as seedling growth, tissue development and responses to environmental stresses [122]. Similarly, the potential role of *GRAS* TF from sweet potato (*Ipomoea batatas* L.) in stress responses was reported by Zhang et al. [123]. Overall, the understanding of the GRAS protein family has greatly advanced. *GRAS* TFs have been shown to play multifunctional roles in the regulation of plant growth, nodulation, microsporogenesis, fruit ripening, tillering, flowering, seed germination, arbuscular mycorrhizal (AM) symbiosis and resistance to various biotic and abiotic stress factors [124,125]. However, their specific characteristics and biological roles remain only partially understood [125]. Recent studies summarized the multifunctional roles of *GRAS* TFs in plant growth, development and responses to multiple stresses [126,127]. Among the known TF families, only a few have been extensively studied in response to cold and UV-B radiation stress, with *GRAS* TFs standing out as one of them [128]. The multifaceted roles of *GRAS7* in plant development, nodulation and stress tolerance, particularly its ability to increase survival under abiotic stress conditions and to affect seed production, establish it as a promising target for the improvement of crop resilience and productivity in challenging environments (Table 1).

## 7. Zn Finger CCHC Type Proteins

The family of proteins harboring zing finger domains is widely distributed in all living organisms. In this family, proteins with the CCHC-type Zn-finger domain remain relatively understudied [129]. These proteins were initially described in retroviruses like HIV with a key role in the viral lifecycle [130]. CCHC-type Zn-finger proteins have various functions related to DNA recognition, RNA packaging, transcriptional activation, regulation of apoptosis and lipid binding [131]. The CCHC domain is defined by the conserved motif Cys–X_2_–Cys–X_4_–His–X_4_–Cys, where X can be any amino acid. Mutations in the conserved zinc-binding residues, particularly the three Cys or His, result in proteins defective in RNA binding. Through their specialized domain architecture, CCHC-type zinc finger proteins play essential roles in the mediation of protein–protein interactions and the metabolism of nucleic acids [129].

### 7.1. The Role of MtZn-CCHC in Flower Morphology and Seed Size

The function of a CCHC-type Zinc-finger protein in flower morphology and seed size was investigated by Radkova et al. [44]. The gene was isolated based on sequence analysis of a *Tnt1* insertion mutant line of *M. truncatula*, showing similarity to a Zn finger CCHC-type gene (ABE91952.1) [30]. To explore its role, three types of transgenic *M. truncatula* plants were constructed—transcriptional reporter lines carrying *GUS* and *GFP* reporter genes and lines with modified expression of *MtZn-CCHC* [44]. Analysis of *M. truncatula* transcriptional reporter lines showed GUS staining in developing shoots, axillary buds and the vascular system of leaves and stipules. In overexpression lines, a strong GFP signal was detected in the anthers, with no signal in the stamen holder and or other flower parts. Phenotypic analysis of *MtZn-CCHC^RNAi^* lines revealed reduced seed size, shorter stems and internodes, and disrupted flower morphology. Beyond *M. truncatula*, studies in other species further displayed the role of *Zn-finger CCHC* genes in seed traits. Abd El-Wahab et al. [132] identified single nucleotide polymorphisms (SNPs) associated with seed length and width in fenugreek, where contig sequences linked to these traits showed similarity to the *MtZn-finger CCHC* gene described by Radkova et al. (2019) [44]. In water yam (*Dioscorea alata* L.), Mondo et al. [133] identified *Zn-finger CCHC* genes among several genes affecting plant reproduction. Similarly, a genome-wide association study (GWAS) in African yam bean (*Sphenostylis stenocarpa*) found candidate genes related to seed size with molecular markers for hundred-seed weight (HSW) showing close homology to *MtZn-CCHC* [134]. More recently, Uba et al. [135] conducted a GWAS in Bambara groundnut [*Vigna subterranea* (L.) Verdc.] that identified 17 candidate genes associated with agro-morphological traits, including several with Zn-finger CCHC domains.

### 7.2. MtZn-CCHC—A Gene with Multiple Functions

The heterologous expression of *MtZn-CCHC* in *A. thaliana* was reported by Radkova et al. [45]. Phylogenic analysis revealed that *MtZn-CCHC* clusters closely with genes from *Trifolium pretense* and *G. max*, which share the same type of Zn-finger domain. In transcriptional reporter plants, the reporter gene signal was localized in the shoot apical meristem and the base of siliques, suggesting specific accumulation in tissues with high meristematic activity. This pattern is consistent with findings for *OsCSP1* and *OsCSP2* in rice, which encode RNA-binding proteins containing Zn-finger CCHC domains [136]. In *A. thaliana* RNAi lines, suppression of the endogenous *AtZn-CCHC* gene led to several phenotypic changes: shortened stem, fewer and shorter siliques, reduced seed number per silique and decreased expression of the meristem marker gene *AtSWP* [45]. The *STRUWWELPETER* (*SWP*) gene is known to regulate cell proliferation and shoot meristem development [137]. Similar phenotypes were observed in the mutant *swp* mutants: shorter organs and fewer siliques with reduced seed number. Previous studies by Sasaki et al. [138] and Nakaminami et al. [139] further demonstrated that *AtCSP2* is highly expressed in meristematic and developing tissues, reinforcing the role of Zn-finger CCHC domain proteins in meristem function and organogenesis. The *AtCSP2* gene encodes a glycine-rich protein (GRP) that contains both a Zn-finger CCHC domain and a cold shock domain (CSD), which show the multifunctionality of these proteins. In the study by Radkova et al. [45], in silico analysis of promoter cis-elements in *MtZn-CCHC* and its homolog *AtZn-CCHC* suggested their regulatory roles in meristem activity, seed development and responses to cold stress. Cold treatment of *A. thaliana* plants overexpressing *MtZn-CCHC* resulted in increased transcript levels of *RD29A*, used as a positive control. The *RD29A* gene encodes the Low-Temperature-Induced protein 78 (LTI78), which functions in cold stress signaling mediated by the overexpression of *CBF/DREB1*. Similar responses have been reported for cold shock protein genes, including *AtCSP2* in *A. thaliana*, and *OsCSP1* and *OsCSP2* in *O. sativa*, confirming their regulation by cold stress and developmental signals [136,138]. A study by Sun et al. [140] described a family of genes encoding CCHC-type zinc finger proteins (ZFPs) in *Triticum* sp. The presence of numerous cis-acting elements in the promoter regions of *TaCCHC-ZFP* genes, associated with environmental stress and phytohormone responsiveness, suggest their involvement in multiple signaling pathways. Similarly, Khassanova et al. [129] isolated two *CaZF-CCHC* genes, Ca04468 and Ca07571, from chickpea (*C. arietinum* L.) as potentially important candidates associated with plant responses to drought and dehydration. These findings support the role of *MtZn-CCHC* in shoot meristem development, seed size regulation and cold stress responses, which confirm its function as a multifaceted regulator in plants (Table 1).

## 8. Conclusions and Perspectives

### 8.1. Advances in Functional Genomics for Crop Improvement

The extensive application of functional genomics has profoundly advanced our understanding of the molecular mechanisms underlying plant growth, development and stress responses, particularly in the model legumes *M. truncatula* and *L. japonicus*. This review provides a comprehensive synthesis of the long-term research, elucidating several key regulatory genes and their networks: the epigenetic regulator *HAC1* that governs histone acetylation and modulates chromatin dynamics; F-box protein regulating BCAA homeostasis and protein turnover; the hormone signaling components *LAX3* and *ARF-B3* coordinating auxin transport and signaling; the transcriptional regulator *GRAS7* that influences stress adaptation and developmental regulation; and multiple plant development and performance regulator *Zn finger CCHC*. The translational potential of these findings extends beyond model species to economically important legumes like soybean, alfalfa and clover, paving the way for advancements in stress tolerance, nutrient use efficiency, root architecture and nodulation, reproductive development, and seed yield and size. Functional genomics has revealed mechanisms underlying key processes such as cell cycle regulation and leucine homeostasis, which offer opportunities to improve agriculturally important traits. Optimization of BCAA biosynthesis pathways presents promising opportunities for the improvement of protein quality and development of nutrient-enriched crop varieties. Since BCAAs are synthesized only by plants and are essential for livestock and human nutrition, understanding their regulation by F-box proteins provides a basis for the improvement of the nutritional value of legume crops. Key agricultural traits like nutrient use efficiency and stress tolerance, exemplified by the role of auxin transporters in phosphate and drought responses, are fundamental to ensure global food security. These insights support the development of resilient, high-yielding crops capable of adapting to changing environmental conditions. Moreover, the integration of functional genomics with multi-omics technologies (transcriptomics, proteomics and metabolomics) has provided a holistic view of plant stress responses, which advanced the discovery of novel genetic targets and the capacity to modify key traits, accelerating progress in crop improvement.

### 8.2. Future Directions and Perspectives for Crop Improvement

**Figure 2:**
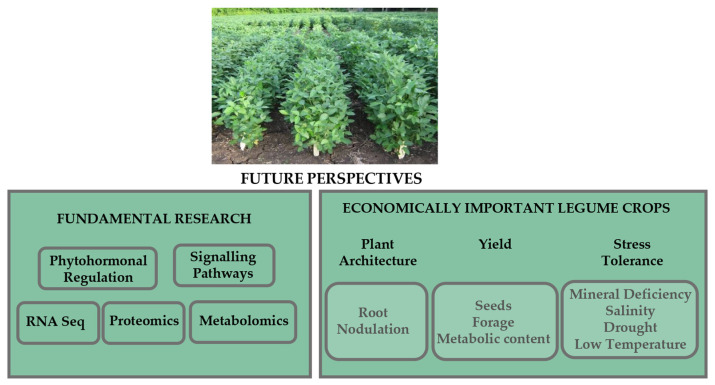
Future perspectives in legumes system biology research for global crop improvement. The image presents an experimental soybean field in Pavlikeny, Bulgaria.

Future research should expand the inclusion of underutilized legume species to broaden genetic resources and support the development of climate-resilient varieties through precise genetic modifications (Figure 2). Investigation of gene network interactions under diverse stress conditions, the use of molecular knowledge to implement sustainable agricultural practices, and the establishment of predictive models for crop improvement using systems biology approaches are essential steps forward. Advancements in functional genomics, combined with emerging technologies and interdisciplinary collaboration, establish a robust foundation to address global food security challenges and foster sustainable agricultural systems. Success in these endeavors will demand coordinated efforts among academic institutions, the agricultural industry and regulatory bodies to effectively translate molecular information into practical scalable solutions. The integration of genomic advancements into breeding programs and modern agricultural practices holds immense potential for the development of resilient, high-yielding crops adapted to changing environmental conditions. A deeper understanding of plant molecular mechanisms, from gene regulation to protein function, provides a powerful platform for driving innovation in sustainable farming practices and securing a stable food supply for future generations.

## Figures and Tables

**Table 1:** Legume genes characterized by functional genomics in Bulgaria. SE, Somatic Embryogenesis; SN, Symbiotic Nitrogen fixation.

Item	Sub-Item		Genes	References
Gene expression	Transcriptional regulation		*HAC1*	[32,33]
			*ARF-B3*	[34,35,36]
			*GRAS7*	[37]
			*Zn-CCHC*	
	Protein degradation		*F-Box*	[38]
Growth and development	Auxin		*LAX3*, *ARF-B3*	[34,35,36,39]
	Amino acid metabolism		*HAC1*, *F-box*	[38,40]
	Cell division		*HAC1*, *F-box*	[33,41]
	SE		*HAC1*, *F-box*, *LAX3*, *ARF-b3*	[33,34,35,36,39,42]
	SN		*HAC1*, *F-box*, *LAX3*, *ARF-B3*, *GRAS7*	[33,34,36,37,39,42,43]
	Organ development	Seed	*F-box*, *LAX3*, *ARF-B3*, *GRAS7*, *Zn-CCHC*	[34,35,36,39,42,43,44,45]
		Flower	*HAC1*, *ARF-B3*, *GRAS7*, *Zn-CCHC*	[32,33,37,44,45]
		Leaf	*F-box*, *LAX3*, *ARF-B3*, *GRAS7*, *Zn-CCHC*	[34,35,36,38,39,42,43,44]
		Root	*F-box*, *LAX3*, *AFR-B3*, *GRAS7*	[34,35,36,39,43,46]
Environmental responses	P nutrition		*LAX3*	[47,48]
	Salinity		*HAC1*, *GRAS7*	[40,43]
	Drought		*GRAS7*	[43]
	Low temperature		*HAC1*, *GRAS7*, *Zn-CCHC*	[40,43,45]

## Data Availability

The data presented in this study are available on request from the corresponding author.
